# Ghrelin signalling in AgRP neurons links metabolic state to the sensory regulation of AgRP neural activity

**DOI:** 10.1016/j.molmet.2023.101826

**Published:** 2023-10-26

**Authors:** Wang Lok So, Jiachen Hu, Lotus Jeffs, Harry Dempsey, Sarah H. Lockie, Jeffrey M. Zigman, Romana Stark, Alex Reichenbach, Zane B. Andrews

**Affiliations:** 1 Monash Biomedicine Discovery Institute and Department of Physiology, Monash University, Clayton 3800, Victoria, Australia; 2 Center for Hypothalamic Research, Department of Internal Medicine, UT Southwestern Medical Center, Dallas, TX, USA; 3 Division of Endocrinology, Department of Internal Medicine, UT Southwestern Medical Center, Dallas, TX, USA; 4 Department of Psychiatry, UT Southwestern Medical Center, Dallas, TX, USA; 5 The Florey Institute of Neuroscience and Mental Health, Mental Health Division, Parkville, Melbourne, Australia

**Keywords:** Growth Hormone Secretagogue Receptor, Photometry, Dopamine, Motivation, Ventral tegmental area, Food intake

## Abstract

**Objective:**

The sensory detection of food and food cues suppresses Agouti related peptide (AgRP) neuronal activity prior to consumption with greatest suppression occurring in response to highly caloric food or interoceptive energy need. However, the interoceptive mechanisms priming an appropriate AgRP neural response to external sensory information of food availability remain unexplored. Since hunger increases plasma ghrelin, we hypothesized that ghrelin receptor (GHSR) signalling on AgRP neurons is a key interoceptive mechanism integrating energy need with external sensory cues predicting caloric availability.

**Methods:**

We used *in vivo* photometry to measure the effects of ghrelin administration or fasting on AgRP neural activity with GCaMP6s and dopamine release in the nucleus accumbens with GRAB-DA in mice lacking ghrelin receptors in AgRP neurons.

**Results:**

The deletion of GHSR on AgRP neurons prevented ghrelin-induced food intake, motivation and AgRP activity. The presentation of food (peanut butter pellet) or a wooden dowel suppressed AgRP activity in fasted WT but not mice lacking GHSRs in AgRP neurons. Similarly, peanut butter and a wooden dowel increased dopamine release in the nucleus accumbens after ip ghrelin injection in WT but not mice lacking GHSRs in AgRP neurons. No difference in dopamine release was observed in fasted mice. Finally, ip ghrelin administration did not directly increase dopamine neural activity in the ventral tegmental area.

**Conclusions:**

Our results suggest that AgRP GHSRs integrate an interoceptive state of energy need with external sensory information to produce an optimal change in AgRP neural activity. Thus, ghrelin signalling on AgRP neurons is more than just a feedback signal to increase AgRP activity during hunger.

## Highlights

• Ghrelin receptors on AgRP neurons integrate hunger with sensory information to optimise the change in AgRP neural activity.
• AgRP ghrelin receptors are required for ghrelin-induced food intake, food-seeking, motivation and AgRP activity.
• AgRP ghrelin receptors mediate the appropriate AgRP inhibitory response to sensory information.
• Ghrelin receptors influence dopamine release in the nucleus accumbens in response to sensory information.
• Peripheral ghrelin injection does not directly increase activity of dopamine neurons in the ventral tegmental area.

## 1 Introduction

Agouti-related peptide (AgRP) neurons within the arcuate nucleus (ARC) of the hypothalamus are critical for regulating appetite, metabolism and preventing the adverse consequences of starvation [1,2]. Artificial AgRP neuronal activation mimics the effects of energy deficit by enhancing food intake and reducing energy expenditure [[3], [4], [5], [6]] and the important role for AgRP neurons in energy balance is underscored by the body weight loss and starvation associated with genetic ablation in adulthood [[7], [8], [9]]. In line with this, AgRP neurons can be classified as hunger-sensing, since photostimulation in ad libitum mice drives a learned operant sequence previously rewarded only in a fasted state [10]. Thus, AgRP neurons are considered the canonical drivers of food intake and metabolism in response to energy deficit and homeostatic need [[11], [12], [13], [14], [15]].

During hunger, hormonal feedback plays an important regulatory role over AgRP neurons. This includes high plasma ghrelin, which increases AgRP activity [16,17], and low levels of leptin and insulin, which reduces their inhibitory input on AgRP neurons [18,19]. However, recent studies using *in vivo* recording techniques in AgRP neurons show these neurons are inhibited by sensory cues of food availability and caloric density [12,20,21]. To maintain a decrease in AgRP neuronal activity following food consumption, feedback from the gut is required to confirm sufficient calorie consumption [[22], [23], [24]]. Thus, the influence of AgRP neurons on energy homeostasis involves the integration of both rapid presynaptic sensory input, as well as neuroendocrine negative feedback associated with food consumption.

The decrease in AgRP neuronal activity in response to food is proportional to the metabolic need of the animal, as well as the caloric density of the sensed food [21,25]. This observation suggests that AgRP neurons integrate interoceptive cues of energy need together with external sensory cues predicting food availability. In many circumstances the balance between internal energy need and food availability in an external environment influences behaviour. For example, pre-emptive AgRP neuronal stimulation drives fed mice to seek food rewards despite the threat of shock [26]. Therefore, interoceptive signals of energy need must influence the salience of sensory inputs onto AgRP neurons. While numerous studies have assessed the nature of the synaptic input on to AgRP neurons [27,28] or the gut-brain feedback coupled to calorie consumption [[22], [23], [24]], the interoceptive signals priming AgRP neuronal responses to sensory input in the fasted state remain unknown.

We hypothesize that such a mechanism should involve neuroendocrine feedback conveying energy need prior to the sensory detection of food or learned food cues. One potential feedback mechanism is an increase in plasma ghrelin during fasting [29] acting through Growth Hormone Secretagogue Receptor (GHSR) on AgRP neurons (AgRP^GHSR^). In many cases the actions of ghrelin phenocopy those of AgRP neurons; 1) ghrelin is a bona fide hunger signal as it elicits an operant behavioural response learned during hunger (fasting) [10,30]; 2) ghrelin increases food intake, motivation and dopamine neural circuits [16,[31], [32], [33]]; 3) ghrelin supresses energy expenditure and lipid utilisation [34,35]; and 4) ghrelin reduces anxiety-like behaviour and increases exploratory behaviour [[36], [37], [38]]. All of the above functions described for ghrelin and/or GHSR signalling have also been linked to AgRP neurons [6,25,[39], [40], [41], [42]] and indeed some show a direct role of GHSR signalling in AgRP neurons on food intake, meal duration, energy expenditure and thermogenesis [[43], [44], [45]]. In this study, we hypothesized that GHSR expression on AgRP neurons is a key interoceptive mechanism integrating energy need with the salience of external sensory detection of food or food cues. To do this, we examined whether GHSR deletion in AgRP neurons affected AgRP neural activity or dopamine release in the nucleus accumbens (NAc) using *in vivo* fibre photometry in response to food or non-food objects.

## 2 Methods

### 2.1 Mice and housing

Mouse experiments were conducted in compliance with the Monash University Animal Ethics Committee guidelines. External environment was maintained under standard laboratory conditions at 23 °C in a 12-hour light/dark cycle with *ad-libitum* access to chow (chow diet, 20% protein 4.8% fat chow diet; Specialty Feeds, Western Australia) and water. Male mice on a C57BL/6J background (8 weeks or older) were used for experimentation and group-housed unless destined for use in fibre photometry. *Agrp-ires-cre* mice [46] were obtained from Jackson Laboratory *Agrp*^*tm1(cre)Low/J*^ (stock no. 012899) and bred with floxed-GHSR1 mice [47] to delete GHSR from AgRP neurons. For *in vivo* photometry studies, *Agrp*^*cre/wt*^*::Ghsr*^*wt/wt*^ mice were used as control animals (designated as AgRP GHSR WT) and A*grp*^*cre/wt*^*::Ghsr*^*flox/flox*^ mice were used as experimental mice (designated as AgRP GHSR KO) to allow for cre-dependent expression of GCaMP6s specifically in AgRP neurons. For food intake and anxiety-like behavioural studies, *Agrp*^*wt/wt*^*::Ghsr*^*flox/flox*^ mice were used as control animals (designated as AgRP GHSR WT) and A*grp*^*cre/wt*^*::Ghsr*^*flox/flox*^ mice were used as experimental mice (designated as AgRP GHSR KO). *Dat-ires-cre* mice [48] from Jackson Laboratory (B6.SJL-*Slc6a3*^*tm1.1(cre)Bkmn*^/J; stock no. 006660) were used to examine the effect of peripheral ghrelin administration on dopamine neural activity in the Ventral Tegmental Area (VTA).

### 2.2 Feeding behaviour studies

To accurately assess food intake and food motivation we used home cage Feeding Experimental Devices 3 (FED3) [49] placed in home cages of individual housed mice. Male mice, aged between 8 and 12 weeks, were allowed to freely collect chow diet pellets (Energy [kcal/g] from protein 24.1%, fat 10.4, carbohydrate 65.5; 5TUM, TestDiets, CA, USA) under a fixed ratio (FR) of 1 for 7 days, 24 h per day, in which 1 poke into an active (left) nose poke port delivered 1 pellet. This led to stable and accurate responding to the active poke and mice collected on average 200 20 mg (4 g) chow pellets per day. To measure food intake in response to ghrelin (1 mg/kg) or saline, mice were injected intraperitoneally (ip) during the light phase when the natural feeding drive is low and chow pellet consumption was measured on an FR1 schedule and recorded with FED3s for 90 min. At least 3 days following IP ghrelin FR1 feeding experiments, FED3s were programmed to deliver chow pellets overnight on an FR 3 schedule followed by an FR5 schedule where mice are required to nose poke 3 or 5 times to collect a single pellet respectively. Following this, mice underwent a progressive ratio (PR) schedule session to measure ghrelin-induced motivation (ip ghrelin 1 mg/kg or saline). The PR session measures motivation as the number of nose pokes required to collect a pellet progressively increases with each pellet delivered. The 90-minute PR session was based on a Richardson Roberts schedule where the number of pokes required to obtain a reward increased in the following pattern; 1, 2, 4, 6, 9, 12, 15, 20, 25, 32, etc [50] and all sessions were conducted during the light phase when natural motivation levels are low. In the buried food finding test we measured the latency to find a familiar buried palatable froot loop (*Kellog's cereal*). Mice were fasted for 6 h prior to beginning the test and all tests were performed within the early dark phase, when mice are normally feeding. For testing, a froot loop was buried approximately 2 cm beneath bedding in a test cage. The latency to find the froot loop, defined as first contact, was videoed and the latency was calculated. Mice had 6 min to complete the task.

### 2.3 Stereotaxic surgery

Stereotaxic surgeries were performed on adult males at least 10 weeks of age. Mice were anaesthetised with 2–3% isoflurane (Baxter, Australia) and injected with Metacam (5 mg/kg, Boehringer-Ingelheim) prior to placing into a heat pad-mounted (37C) stereotaxic frame (Stoelting/Kopf). For GCaMP6s expression in AgRP neurons, cre-dependent AAV9-hSyn-FLEX-GCaMP6s-WPRE-SV40 (∼2.0 × 10^12^ vg/ml; Addgene #100845) was bilaterally injected into the ARC (Coordinates: 1.6 mm anterior-posterior; 0.2 mm lateral; −5.8 mm dorsoventral from the surface of the brain, 200nl/side infused at a rate of 40 nl/min and allowed to rest for 5 min post-infusion). For experiments using GCaMP6s in *Dat-ires-cre* mice, cre-dependent AAV9-hSyn-FLEX-GCaMP6s-WPRE-SV40 (∼2.0 × 10^12^ vg/ml; Addgene #100845) was injected unilateral into the VTA (Coordinates: 3.1 mm anterior-posterior; 0.5 mm lateral; −4.4 mm dorsoventral from the surface of the brain, 200nl/side infused at a rate of 40 nl/min and allowed to rest for 5 min post-infusion). For experiments involving dopamine release, mice were unilaterally injected with the non-cre dependent dopamine biosensor – GRAB-DA (∼2.0 × 10^12^ vg/ml, WZ Biosciences, MD, USA; YL10012-AAV9: AAV-hSyn-DA4.3) in the nucleus accumbens (bregma 1.2 mm, 0.5 mm lateral, −4.8 mm from the surface of the brain; 200 nl infused at a rate of 40 nl/min and allowed to rest for 5 min post-infusion). Ferrule-capped fibres (400 μm core, NA 0.48 Doric, MF1.25 400/430–0.48) were placed above the site of injection and secured with dental cement (GBond, Japan).

In an additional experiment, *Agrp*^*cre/wt*^*::Ghsr*^*wt/wt*^ and A*grp*^*cre/wt*^*::Ghsr*^*flox/flox*^ mice were injected with both cre-dependent AAV9-hSyn-FLEX-GCaMP6s-WPRE-SV40 (as above) and cre-dependent AAV5-hSyn-DIO-hM3D (Gq)-mCherry (∼2.0 × 10^12^ vg/ml; Addgene #44361) bilaterally injected into the ARC to test the functional capacity of AgRP GHSR KO to increase food intake to a ghrelin independent signal. Mice were given a recovery period of two weeks after surgery and to allow for viral transduction before experimentation.

### 2.4 Fibre photometry

All recordings were performed using connectorized LEDs, LED drivers, Fluorescent Mini Cubes, rotary joints and photoreceivers (model no. 2151; Newport) from Doric Lenses (Quebec, Canada). These optical components were controlled by a Tucker Davis Technologies (TDT) RZ5P processor using TDT Synapse software for demodulation, low-pass filtering (4Hz) and data acquisition. Two excitation wavelengths were used to deliver 465 nm and 405 nm, in which the 465 nm wavelength reported a Ca^2+^ dependent GCaMP6 signal or dopamine-specific GRAB-DA signal and the 405 nm wavelength served as an isosbestic control for motion artifact. The isosbestic wavelength is where excitation is independent from intracellular Ca^2+^ (GCaMP6) or extracellular dopamine release (GRAB-DA).

Behavioural events were marked by the researcher using Synapse during recordings or using Open Scope software (Tucker–Davis Technologies) after recordings to precisely align behavioural events with neural activity data. For data processing, custom written python codes extracted and down sampled 465 nm and 405 nm signals to every 100 ms (codes available at Github). These down sampled 465 nm and 405 nm signals were then used to calculate ΔF/F using the following equation (F465 nm-F405 nm/F405 nm) to correct for photobleaching of the signal and movement artefacts. Photobleaching was negligible due to the short experimental time frame (∼30–45 min).

For data analysis, z-score normalisation was used for each behavioural event (i.e. pellet drop, injection) where the degree of change is relative to a predefined baseline period. Z-score normalisation used the following formula; z = (F-Fμ)/Fσ, where F is the signal and Fμ and Fσ are the mean and standard deviation of the baseline signal. A z-score highlights the number of standard deviations a data point is away from the baseline mean.

### 2.5 Fibre photometry behavioural experiments

Mice had either ad libitum access to chow diet (fed state) and were fasted overnight for 14 h (fasted state). Mice were habituated to the photometry setup prior to experimentation and on the day of experiments, mice had 10 min to acclimatize before starting recordings. In 2-min intervals, a small wooden dowel (novel food object; chewing control) was dropped into the enclosure followed by a chow pellet and peanut butter (PB) pellets. PB pellets were created by dropping melted Reese's peanut butter chips onto parafilm with a 10 ml syringe, such that all PB pellets were an approximately uniform size (average weighed 50 mg). This ensures an assessment of AgRP activity or dopamine release to PB consumption is standardised and any differences are related to genotyping rather than total calories consumed and mice always consumed the PB pellet in all trials. For these studies, we did not perform chow presentation as fed mice showed little interest in approaching or consuming chow food during the recording periods. For experiments involving IP injection of saline or ghrelin (1 mg/kg; BOC Sciences) or CNO (1/mg/kg; Sigma Aldrich, in saline), baseline GCaMP6s activity was measured for 15 min prior to injection, at least 25 min after injection without food and for a further 10 min after food presentation (GCaMP6s studies). For GRAB-DA studies, baseline dopamine release was measured for 15 min prior to injection before exposure to PB pellets. All photometry recordings were conducted during the early light phase.

### 2.6 Anxiety-like and exploratory behaviours

Ad libitum or overnight fasted AgRP GHSR WT and KO mice were tested in elevated plus maze (EPM), light dark box (LD box) or open field arenas. Each trial was video tracked and analysed with Ethovision (Noldus Information Technology; Wageningen, the Netherlands) to quantify mouse performance within defined regions of behavioural arenas. The order in which the mice were assessed was counterbalanced between each test to minimise order effects. The arenas were thoroughly cleaned with 70% ethanol between each trial. A minimum of 1 week was required between repeated exposures of the same mouse to the same arena. Each test was performed during the early light phase to align with the time of GCaMP6 or GRAB-DA recordings.

### 2.7 Immunohistochemistry

The efficiency of viral transduction was confirmed by immunohistochemical detection of GFP expression in the ARC of *Agrp*^*cre/wt*^*::Ghsr*^*wt/wt*^*,* A*grp*^*cre/wt*^*::Ghsr*^*lox/lox*^ or the VTA of *Dat*^*cre/wt*^ mice. Mice were perfused and fixed with 0.05M phosphate buffered saline (PBS) followed by 4% paraformaldehyde (PFA) in PBS. Brains were immediately removed and post-fixed for 24 h in 4% PFA at 4 °C before being transferred to 30% sucrose in 0.1M PB solution for 2–5 days. Brains were sectioned at 30 μm on a cryostat (Leica CM1800). Sections were collected in sets of 4 and stored in 24-well plates suspended in cryoprotectant at −20 °C. For staining, sections were washed in 0.1M PBS (3 × 10 m min) and then blocked for 60 min in 4% normal horse serum in PBS + 0.3% Triton-X. Sections were incubated in primary antibody (chicken anti-GFP; ab13970; abcam) diluted 1:1000 in blocking solution overnight at 4 °C. The next day, sections were washed in 0.1M PB (3 × 10 min) and incubated with secondary antibody (goat anti-chicken; AB_2337390; Jackson ImmunoResearch) at a dilution of 1:500 in 0.1M PB for 2 h at room temperature. Following a final wash, sections were mounted onto SuperFrost slides (Thermo Fisher Scientific) with VECTASHIELD antifade mounting medium with DAPI (Vector Labs). Post-curing, slides were then sealed with nail polish and stored at 4 °C. Sections were imaged under an upright fluorescence microscope (Zeiss Axio Imager 2; Zeiss, Germany). Coronal brain slices were outlined and tiled with an EC Plan-NEOFLUAR 5x objective (0,15NA, air), while focal images were captured through an EC Plan-NEOFLUAR 10x objective (0,3NA, air). Prior to tiling acquisition, exposure, gain and offset were adjusted to the brightest spot on the sample. Care was taken to ensure no pixels were oversaturated.

### 2.8 Statistical analysis

Data are represented as mean ± SEM and all statistical analyses were performed using GraphPad Prism for MacOS X. Two-way ANOVAs (with repeated measures as appropriate) and post hoc tests were used to determine statistical significance. Data were tested for normality using a Kolmogorov–Smirnov test. A two-tailed Student's t-test was used when appropriate, as indicated in figure legends. *p* < 0.05 was considered statistically significant.

## 3 Results

### 3.1 AgRP^GHSRs^ regulate food intake and motivation and AgRP neuronal activity

**Figure 1:**
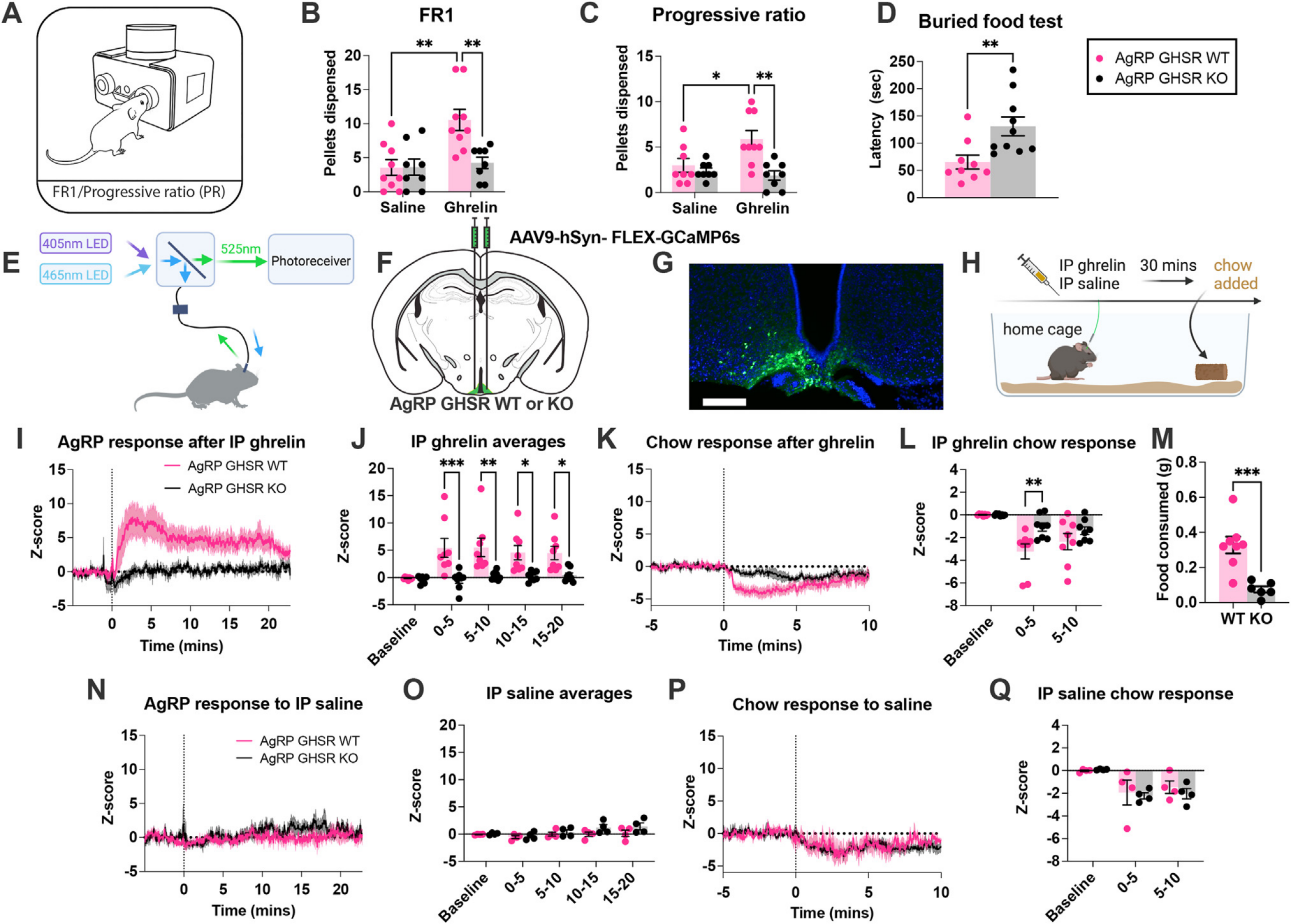
**Deletion of GHSR in AgRP neurons affects food intake, motivation and AgRP neuronal activity.** (A) Schematic representation of a FED3 device used for home cage assessment of food intake, via (B) a fixed ratio 1 (FR1) schedule (WT n = 9; KO n = 8), or (C) food motivation using a progressive ratio (PR) schedule (WT n = 8; KO n = 8). (D) The latency to find a hidden Froot Loop was significantly longer in mildly fasted (6 h) AgRP GHSR KO mice compared to WT mice (WT n = 9; KO n = 10). (E) Schematic representation of a photometry setup, with GCaMP6s excitation at 465 nm and an isosbestic control (405 nm). (F) Schematic of AAV9-hSyn-FLEX-GCaMP6s injection into the ARC of AgRP cre mice. (G) GCaMP6s expression in the ARC of AgRP cre mice; scale bar 100 μm. (H) Schematic experimental timeline of photometry experiments created with BioRender.com. (I) The averaged Z-score of AgRP neuronal responses to IP ghrelin aligned to injection at time = 0 s (WT n = 8; KO n = 8) and (J) averaged Z-score time bins between 0 and 5, 5–10, 10–15 and 15–20 min (WT n = 8; KO n = 8). (K) AgRP neuronal response to chow consumption in AgRP GHSR WT and KO mice (WT n = 8; KO n = 8) with (L) average Z-score time bins showing a greater fall in AgRP activity in WT, compared to KO mice. (M) 1hr food intake measured during the IP ghrelin photometry recordings in WT and KO mice (n = 8 WT and KO). The averaged Z-score response (N), or the time binned responses (O), to IP saline was not different in WT compared to KO mice (n = 4). In response to chow diet consumption, there was no difference in the averaged Z-score over time (P) or in 5-minute time bins (Q). Data +/− SEM. Dotted lines in I and N represent the time of injection and in K & P represent time chow was placed into the cage. Two-way ANOVA with post hoc Sidak's multiple comparisons (B, C, J, L) or students t -test (D, M). ∗p < 0.05, ∗∗p < 0.01, ∗∗∗p < 0.001. For a detailed description of statistics see statistical table 1.

To demonstrate the functional effects of GHSR deletion in AgRP neurons, we examined ghrelin-induced chow pellet intake and motivation using FED3 operant devices (Figure 1A) [49]. Although ip ghrelin (1 mg/kg) significantly increased chow consumption over 90 min in a FR1 feeding schedule in AgRP GHSR WT mice, it failed to induce chow pellet intake in AgRP GHSR KO mice (Fig 1B). An analysis of feeding behaviour during the FR1 experiment revealed ghrelin significantly reduced pellet retrieval time (main effect of treatment) and affected the interpellet interval (significant interaction) (Sup Figure 1C–E). AgRP neuronal stimulation and ip ghrelin both increase motivated food seeking using PR schedules [6,51], therefore we injected ip ghrelin (1 mg/kg) to test whether AgRP^GHSRs^ are necessary for motivated chow food seeking using a PR. IP ghrelin significantly increased the number of pellets consumed during a PR schedule in WT but not KO mice (Fig 1C). During the PR experiments we observed no significant differences in the latency to collect 2 pellets, pellet retrieval time or the interpellet interval (Sup Figure 1F–H), due to the increasing number of unrewarded pokes during the PR. To further functionally validate the loss of GHSR on AgRP neurons, we measured the latency to find a Froot Loop reward in a buried food finding task. After 6-hour fast AgRP GHSR KO mice required twice as much time as WT controls to find the known food reward (Fig 1D).

To show that AgRP^GHSR^ are required for ghrelin to increase AgRP neuronal activity, we used an *in vivo* photometry approach in AgRP GHSR WT and KO mice. IP ghrelin injections rapidly increased AgRP neuronal activity in WT but not KO mice (main effect of genotype), with significant differences in averaged Z-scores at 5-minute time bins after injection (Figure 1I–J). Moreover, IP ghrelin significantly increased AgRP activity when compared to IP saline in WT mice (Sup Fig 1I-L). The subsequent response to chow consumption 20 min after ghrelin injection caused a greater suppression of AgRP neuronal activity from 0 to 5 min in WT compared to KO mice and no significant difference was observed from 5 to 10 min (Fig 1K-L). This coincided with greater chow consumption in AgRP GHSR WT mice during the recording period (Fig 1M). No genotype differences in AgRP neuronal activity were observed in response to IP saline and chow presentation (Fig 1N-O), although chow presentation suppressed AgRP activity (main effect of time; Fig 1P-Q). Finally, co-transduction of AgRP neurons with hM3Gq DREADD and GCaMP6s revealed that CNO equally stimulated AgRP activity in both AgRP GHSR WT and KO mice (main effect of time; Sup Figure 1A–B). GHSRs are Gq protein coupled receptors that activate downstream phospholipase C signalling and the hM3Dq DREADD also utilises a similar Gq protein coupled receptor pathway [52]. Thus, the ability of hM3Dq DREADDs to activate AgRP neurons equally in AgRP GHSR WT and KO mice shows that downstream receptor signalling was not affected. We did not use neuroanatomical approaches to examine the loss of GHSRs on AgRP neurons. Collectively, our functional studies illustrate that our genetic approach to delete GHSR in AgRP neurons prevents ghrelin-induced food intake, motivation and AgRP neuronal activity but does not affect neuronal excitability, confirming that this is a useful model for further studies.

### 3.2 AgRP^GHSRs^ influence AgRP neural activity in response to sensory information

**Figure 2:**
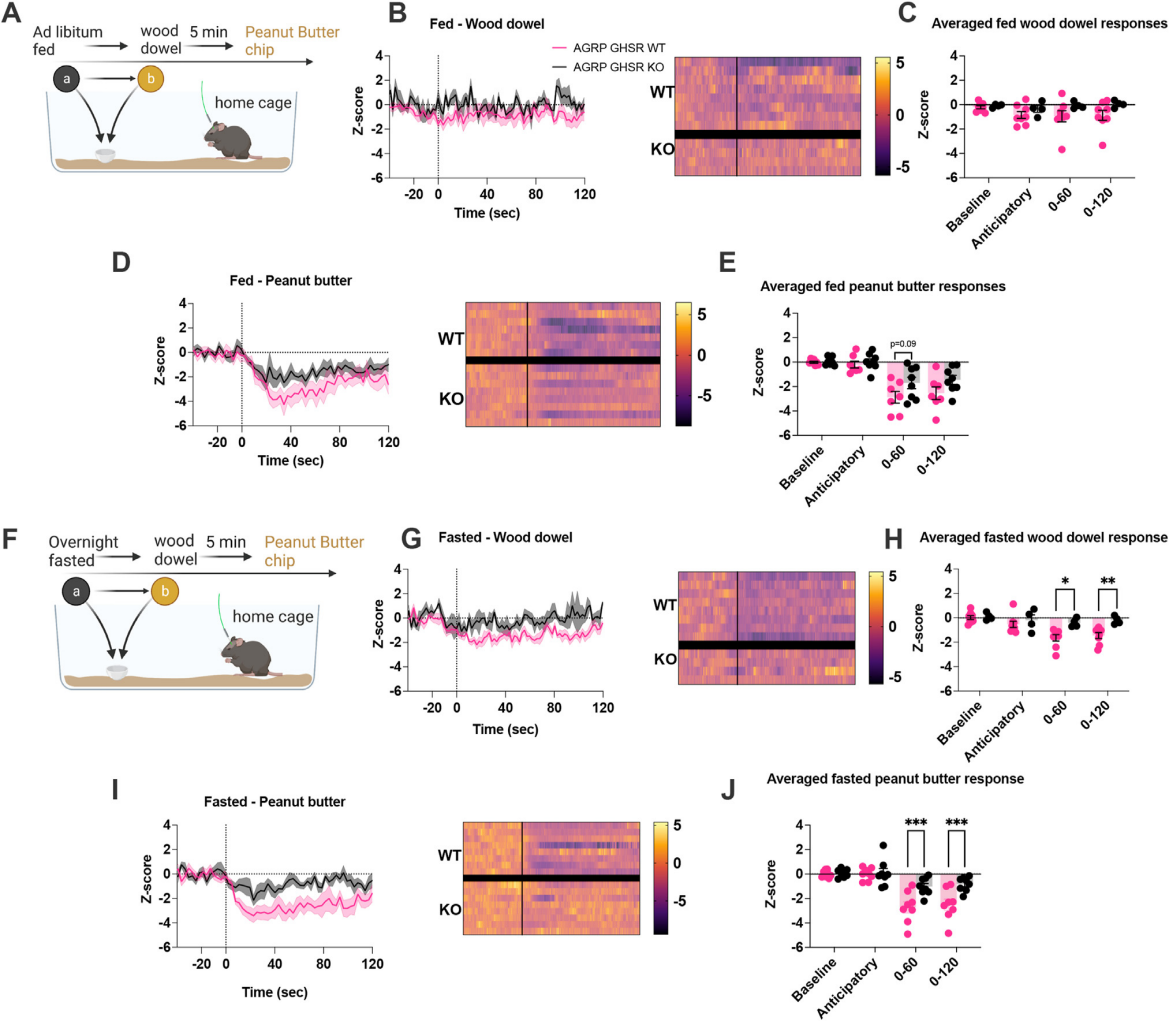
**Deletion of GHSR in AgRP neurons attenuates fasting-induced response to a wood dowel and peanut butter chips.** (A) schematic showing the experimental timeline (created with BioRender.com). (B) Average Z-score traces of AgRP neuronal activity in response to a wooden dowel aligned to first contact of the nose to the object with heat maps (WT n = 8, KO n = 4) and (C) the following time averaged bins in baseline (−40 to −20 s), anticipatory (−20 to 0 s), the first minute (0–60 s) and 2 min (0–120 s) (WT n = 8, KO n = 4). (E) Average Z-score traces in response to peanut butter aligned to first contact with heat maps (D) and the following time averaged bins in baseline (−40 to −20 s), anticipatory (−20 to 0 s), the first minute (0–60 s) and 2 min (0–120 s) (WT n = 7, KO n = 8). (F) Assessment of AgRP neuronal activity in overnight fasted AgRP GHSR WT or KO mice to a wooden dowel and peanut butter as schematically represented (created with BioRender.com). (G) Average Z-score traces in response to a wooden dowel aligned to first contact of the nose to the object and heat maps and (H) the following time averaged bins in baseline (−40 to −20 s), anticipatory (−20 to 0 s), the first minute (0–60 s) and 2 min (0–120 s) (WT n = 8, KO n = 4). (I) Average Z-score traces in response to peanut butter aligned to first contact and (J) the following time averaged bins in baseline (−40 to −20 s), anticipatory (−20 to 0 s), the first minute (0–60 s) and 2 min (0–120 s). Data +/− SEM. Dotted lines in B, D, G, I represent first contact. Two-way ANOVA with post hoc Sidak's multiple comparisons (H, J). ∗p < 0.05, ∗∗p < 0.01, ∗∗∗p < 0.001. For a detailed description of statistics see statistical table 1.

Sensory detection of food and/or food-specific cues rapidly inhibit AgRP neurons in a manner that is scaled to the metabolic need of the animal and caloric availability of the food or food-predicting cue [21,24,25]. This highlights that AgRP neurons rely on both internal and external information to correctly respond to food and/or cues. While calorie consumption provides AgRP neurons the opportunity to update and encode caloric value with external sensory cues via gut-brain feedback [[22], [23], [24]], exactly how internal cues of metabolic need integrate with external sensory cues to influence AgRP activity remains unknown. To test if GHSRs on AgRP neurons influence AgRP activity in response to external sensory information, we expressed GCaMP6s in both AgRP GHSR WT and KO mice. Small equally-sized PB (50 mg) pellets were used as a high-caloric value food item and a wooden dowel was used as a chewing control item. In the fed state, the introduction of a wooden dowel resulted in a difference in AgRP activity between genotype (main effect of genotype), although no specific differences were identified by post hoc analysis (Figure 2B–C). The presentation of PB pellets to ad libitum fed mice significantly affected AgRP activity (main effect of time) and genotype (main effect of genotype), with post hoc analysis indicating a trend for an attenuated suppression in average Z-score for 0–60 s in KO compared to WT mice (Figure 2D–E).

In fasted mice, the introduction of a wooden dowel resulted in lower AgRP activity (main effect of time), which was attenuated in KO mice across all time bins examined (main effect of genotype). Moreover, post hoc analysis revealed significantly less suppression of AgRP neuronal activity in KO mice at 0–60 and 0–120 min after presentation of a wooden dowel (Figure 2G–H). The presentation of equally-sized PB pellets, to ensure equal caloric consumption across genotypes and trials, resulted in a difference in AgRP activity over time (main effect of time), genotype (main effect of genotype) and difference between genotypes over time (interaction time x genotype; Figure 2I–J). Post hoc analysis demonstrated the fall in AgRP activity in KO mice at 0–60 s and 0–120 s was significantly attenuated compared to WT mice (Fig 2J). Importantly, no differences in anxiety-like or exploratory behaviour were detected between AgRP GHSR WT and KO mice in EPM, LD box or Open Field tests under ad libitum fed or fasted metabolic states (Sup Figure 2 and Sup Fig 3). In addition, we recorded approach time during AgRP neuronal recording sessions and observed no significant effects on approach time to chow food after ghrelin injection, or to PB pellets or wooden dowel in the fed or fasted state (Sup Fig 4A-C). These data suggest that the attenuated fall in AgRP activity to PB pellets or wooden dowel in KO mice was not caused by altered anxiety-like behaviour, an important consideration as both AgRP neurons and the ghrelin system play a role in stress and anxiety-like behaviour [[36], [37], [38],^41^,^42^].

### 3.3 AgRP^GHSRs^ influence ghrelin-induced NAc dopamine release

**Figure 3:**
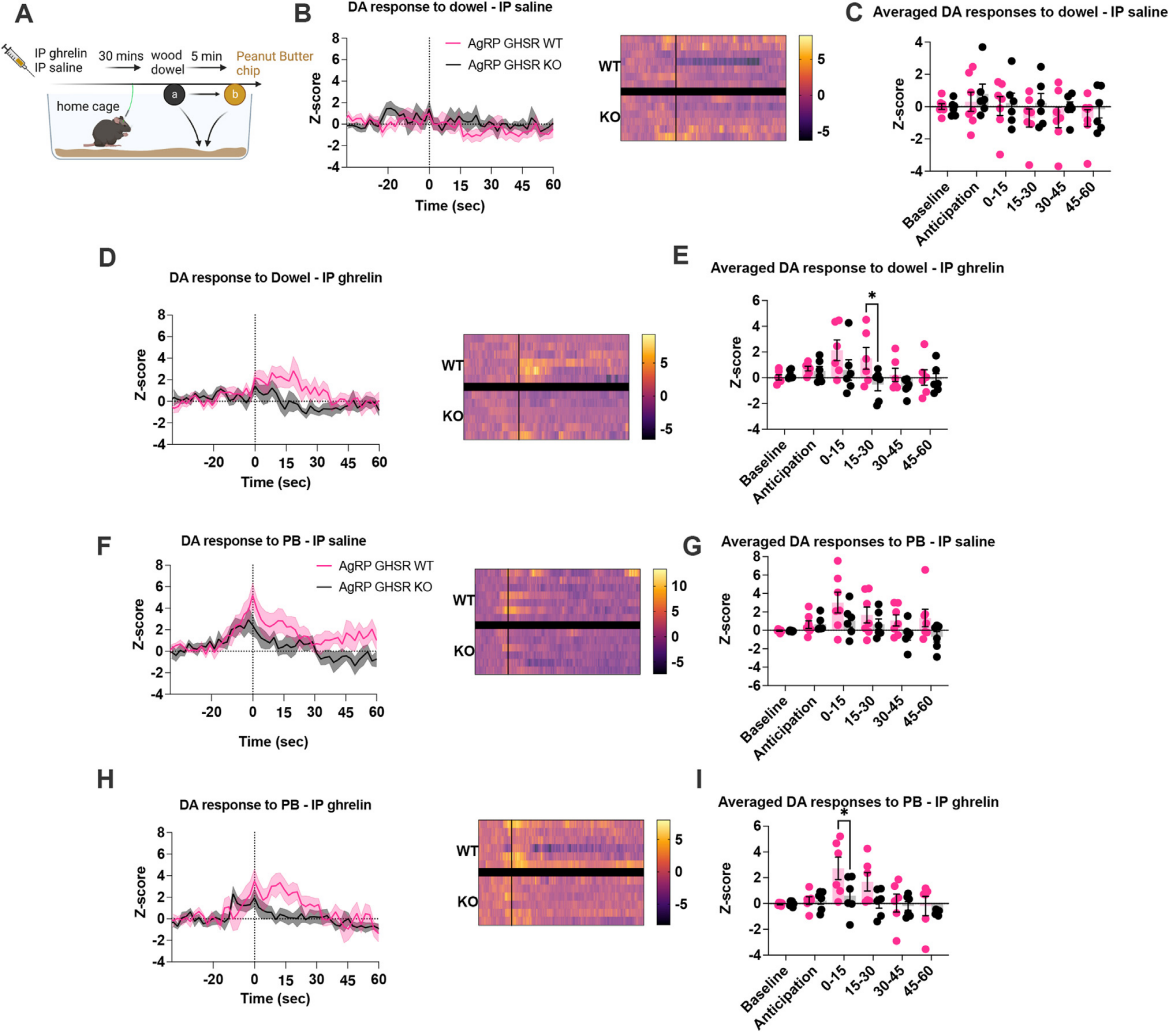
**Deletion of GHSR in AgRP neurons affects ghrelin-induced changes in dopamine (DA) release in the nucleus accumbens.** (A) Schematic of the experimental timeline created with BioRender.com, where mice expressing GRAB-DA in the nucleus accumbens were injected with either ghrelin or saline and presented with a wooden dowel followed by a peanut butter chip (PB). (B) Averaged Z-score traces of dopamine release in the NAc in response to a wooden dowel following IP ghrelin with heat maps (WT n = 7, KO n = 6) or (D) saline with heat maps (WT n = 7, KO n = 6) and respective time binned averages (C and E) organised into baseline (−40 to −20 s), anticipatory (−20 to 0 s) periods and 15-second periods after PB/dowel (0–15, 15–30, 30–45, 45–60). (F) Averaged Z-score traces of dopamine release to PB following IP ghrelin (WT n = 7, KO n = 6) or (H) saline (WT n = 6, KO n = 6) with their respective time bin averages (G and I). Data +/− SEM. Dotted lines in B, D, F, H represent first contact to wood dowel or PB. Two-way ANOVA with post hoc Sidak's multiple comparisons (E, I). ∗p < 0.05. For a detailed description of statistics see statistical table 1.

Previous studies highlight that AgRP neurons influence the development of dopamine neuroplasticity [53] and dopamine release in the NAc [39]. Indeed, impaired metabolic sensing in AgRP neurons reduces dopamine-driven motivation during fasting and reduces striatal dopamine release in response to palatable foods or during operant sucrose seeking [25]. Thus, we used GRAB-DA sensors to assess the impact of sensory cues (wooden dowel, PB pellets) on dopamine release in response to ip saline and ghrelin in AgRP GHSR WT and AgRP GHSR KO mice. Ip injection of saline did not alter NAc dopamine release in response to a wooden dowel in either genotype (Figure 3B–C), however, ip ghrelin resulted in significantly elevated dopamine release in response to a wooden dowel over time. This effect was significantly greater in WT mice (Figure 3D–E). Post hoc analysis indicating dopamine release has significantly lower average Z-score in KO mice 15–30 s after dowel presentation (Fig 3E). In WT mice, ip ghrelin significantly increased dopamine release to a wooden dowel when compared to ip saline injected mice (Sup Fig 5C-D). PB pellet presentation after ip saline results in a significant elevation of dopamine release over time (main effect of time) and this is greater in WT mice (main effect of genotype; Fig 3G), although no specific differences were identified by post hoc analysis (Fig 3G). In contrast, NAc dopamine release after ip ghrelin was significantly reduced in AgRP GHSR KO compared to WT mice at 0–15 s after PB pellet presentation, as assessed by post hoc analysis (Fig 3I). In response to PB presentation, ip ghrelin produced no additional increase in dopamine release after ip saline in WT mice (Sup Fig 5A-B).

Next, we assessed whether GHSR signalling in AgRP neurons influences NAc dopamine to sensory cues (wooden dowel and PB pellets) under fasted conditions, since fasting and energy deficit is a strong driver of ghrelin secretion into the bloodstream [29]. The presentation of a wooden dowel (Sup Fig 6C) or PB pellets (Sup Fig 6E) to ad libitum fed WT and KO mice affected dopamine release over time (main effect of time) although no significant difference between genotypes were observed (Sup Fig 6C, 6E). Similarly, a wooden dowel or PB pellet affected dopamine release over time (main effect of time) with no significant difference between genotypes (Sup Fig 6G, 6I). These studies highlight that AgRP^GHSRs^ do not affect NAc dopamine release in response to fasting.

### 3.4 IP ghrelin does not directly regulate VTA dopamine neurons *in vivo*

**Figure 4:**
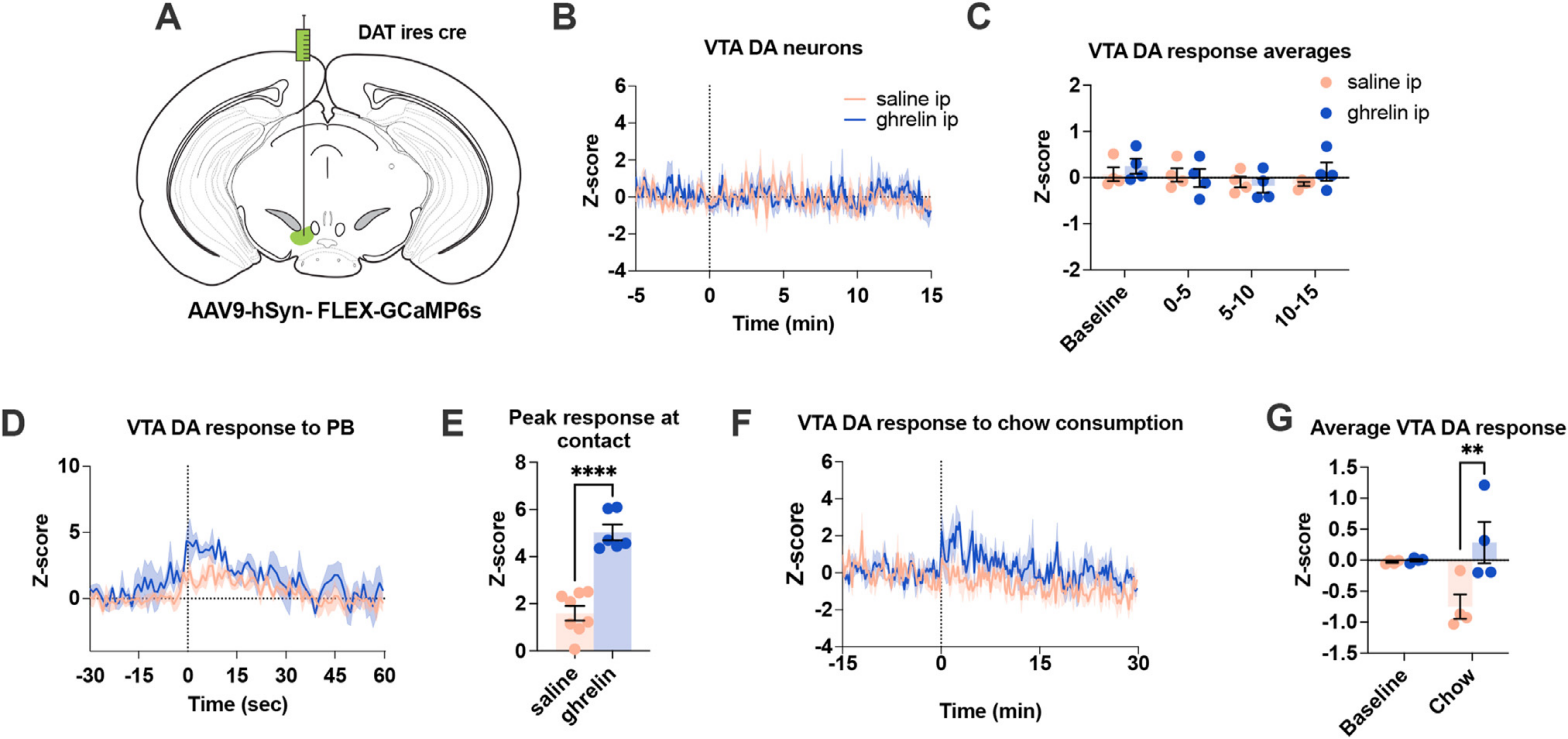
**Ghrelin-induced changes in VTA dopamine neurons.** (A) Schematic showing the approach to record from DA neurons after injection of AAV9-hSyn-FLEX-GCaMP6s in the ventral tegmental area (VTA) in dopamine transporter (DAT) ires cre mice. (B) Average Z-score VTA DA response to an injection of ghrelin or saline (saline & ghrelin n = 4; dotted line represents time at injection) with (C) 5 min time binned data. (D) VTA dopamine neuronal activity at contact with PB (saline n = 4, ghrelin n = 3), with (E) the maximum Z-score response at contact to after saline or ghrelin injection (saline n = 8 contacts from n = 4 mice; ghrelin n = 6 contacts from n = 3 mice). (F) Average Z-score VTA DA response to chow consumption (saline n = 3, ghrelin n = 4; dotted line represents time when chow was placed in cage). (G) The averaged 30-minute Z-score response from the beginning of chow consumption after saline or ghrelin injection (saline n = 3; ghrelin n = 4). Data +/− SEM. Dotted lines in B, D, F represent first contact to PB or chow. Two-way ANOVA with post hoc Sidak's multiple comparisons (H, J). ∗p < 0.05, ∗∗p < 0.01, ∗∗∗p < 0.001, ∗∗∗∗p < 0.0001. For a detailed description of statistics see statistical table 1.

A large number of studies suggest that ghrelin increases motivation and dopamine release by targeting the VTA [31,33,[54], [55], [56], [57], [58]], however the reduced dopamine release after ip ghrelin to both wooden dowel and PB pellets in AgRP GHSR KO suggests a novel role for GHSR signalling in AgRP neurons. To explore how ip ghrelin influences VTA DA neural activity, we used *in vivo* photometry with GCaMP6s delivered via injection of an AAV into the VTA of DAT-ires-cre mice (Figure 4A) to directly visualize the activity of the VTA dopaminergic neurons. Surprisingly, ip ghrelin injection had no effect on the population activity of VTA dopamine neurons (Figure 4B–C). However, ip ghrelin injection acutely increased the population activity of VTA DA neurons to subsequent presentation of PB pellets or chow 10–20 min after injection (Figure 4D–G). This suggests that peripheral ghrelin does not directly regulate VTA dopamine neural activity but rather acts on upstream neural circuits that provide input to the VTA.

## 4 Discussion

In this study, we explored whether AgRP^GHSRs^ integrated interoceptive energy need with the sensory detection of food (PB) and a non-food object (wooden dowel). We found that deleting GHSRs from AgRP neurons impaired ghrelin-induced chow intake, food finding, food motivation and AgRP neuronal activity, confirming the functional GHSR deletion in AgRP neurons from KO mice. These results are supported by studies showing that AgRP neurons are an important target for plasma ghrelin. For example, the majority of AgRP neurons contain GHSRs [59] and AgRP neurons are largely responsible for the ability of ghrelin and fasting to increase food intake and affect energy expenditure [44,[60], [61], [62], [63]]. Further, food deprivation-induced discrimination studies [10,30] show AgRP neurons sense hunger whereas plasma ghrelin signals hunger. Thus, GHSR receptors on AgRP neurons are a critical component of the negative feedback actions of ghrelin on hunger.

However, our results show that AgRP^GHSRs^ influence AgRP neuronal responses to external sensory cues, suggesting ghrelin signalling on AgRP neurons is more than just a feedback signal of hunger. Importantly, the suppression in AgRP activity after the introduction of sensory cues (food – PB, object - wooden dowel) was significantly attenuated in fasted, but not fed, AgRP GHSR KO mice. These results are in line with previous studies demonstrating that AgRP inhibition to food requires the inherent ability of AgRP neurons to metabolise and sense changes in glucose [25]. Thus, our results suggest that AgRP^GHSRs^ are required to integrate the interoceptive energy state with external sensory information to produce the optimal response in AgRP neural activity. Interestingly, Liver-Expressed Antimicrobial Peptide (LEAP2) is an endogenous inverse agonist of the GHSR [64], which hyperpolarises AgRP neuronal activity and prevents ghrelin-induced activation [65]. Given that hunger suppresses plasma LEAP2 [66], future studies are required to examine how circulating LEAP2 influences the activity of AgRP neurons in response to food and sensory cues predicting food availability.

We have recently suggested AgRP neurons regulate energy balance through a process of energy allostasis, rather than negative feedback to perturbations in homeostasis [1]. Energy allostasis incorporates previous experiences to help predict and prepare for perceived future energy demands prior to an energy deficit. For example, sensory cues cause a greater suppression of AgRP neural activity, prior to food consumption, when these cues were previously associated with calorie consumption [24,27]. Intriguingly, AgRP^GHSRs^ influence both the decrease in AgRP activity to the sensory detection of food and act as hunger signals to increase AgRP activity in response to energy need. Therefore, we suggest AgRP^GHSRs^ maintain energy balance through energy allostasis rather than a negative feedback model of energy homeostasis.

Of note, AgRP neuronal responses to a non-food object (a wooden dowel) were also significantly lower when GHSR expression was lacking in AgRP neurons. Recent studies show that sensory information of non-food related events also rapidly suppress AgRP neuronal activity, including thermal pain, maternal reunion, warm exposure (14C→30C) and cessation of running [15,[67], [68], [69]]. The functional consequences of rapid AgRP inhibition to the sensory detection of non-food related stimuli is unknown, yet our results show it is dependent on metabolic state and AgRP^GHSR^ expression. We hypothesise this may be related to an important role in foraging behaviour since the need to forage is greater when hungry and a foraging individual is likely to encounter both food and non-food related objects or events. Indeed, AgRP neurons and ghrelin play important roles in foraging and foraging-related behaviours such as locomotion, exploration and arousal [70,71] and AgRP neurons alter behaviours based on energy need [72].

We noted that ghrelin did not increase motivation in AgRP GHSR KO mice. These results are consistent with the idea that AgRP neurons increase food motivation and food seeking [6,25,73] and they highlight an essential role for AgRP^GHSRs^ in ghrelin-induced motivation. Although ghrelin is well described to influence food reward and motivation, these effects have been ascribed to actions within the mesolimbic dopamine system based on direct brain injections of ghrelin into the VTA or pharmacological and genetic approaches targeting VTA neural activity [31,33,54,56,57,74]. Thus, our results highlight a new pathway for ghrelin to influence motivation by acting on AgRP^GHSRs^.

The presence of GHSRs on AgRP neurons is also important for normal dopamine release in the NAc in response to the introduction of a wood dowel or PB pellet. While previous studies show that ip ghrelin injection increases dopamine release or turnover in the NAc [56,75], the exact site of action was unknown. Our studies directly implicate a role for AgRP^GHSR^ in mediating ghrelin-induced dopamine release. Indeed, AgRP neurons play an important role in motivated feeding behaviour and engage midbrain dopaminergic neurons in the ventral tegmental area (VTA), as well as influencing dopamine release in the nucleus accumbens (NAc) [39,76]. Recently, we showed that metabolic sensing in AgRP neurons was required to translate internal energy need into increased motivated behaviour [25]. An impairment in metabolic-sensing in AgRP neurons also reduced NAc dopamine release to food rewards or during motivated food seeking and attenuated the ability to learn the caloric value of peanut butter [25]. Thus, AgRP neurons actively transmit an interoceptive ghrelin signal to influence NAc dopamine release. However, it should be noted that no significant differences in NAc dopamine release between WT and KO mice were observed in overnight fasted mice, suggesting other pathways must also be involved in fasted-induced dopamine release, such as the intrinsic metabolic-sensing ability of AgRP neurons [25]. GHSR deletion in AgRP neurons also attenuated dopamine release after wooden dowel and PB pellet presentation in response to ghrelin injection. These results suggest that ghrelin-signaling in AgRP neurons affects the salience of both food and non-food events through changes in NAc dopamine release. Again, this may be related to optimal foraging behaviour since foraging involves the exploration and interaction with food and non-food objects/events and dopamine release in the NAc [77].

Interestingly, we did not observe a direct effect of ghrelin injection on VTA dopamine population activity although ghrelin potentiated dopamine neural activity to PB or chow presentation. The lack of a direct effect on VTA dopamine activity is somewhat surprising giving the abundant expression of GHSRs on dopamine neurons [78] and well known role of intra-VTA ghrelin injection on food intake and behaviour [31,33,55,57,79]. Moreover, intra-VTA administration of a GHSR antagonist inhibits the actions of peripherally-administered ghrelin to induce food intake [31] and selective GHSR expression within the VTA improves social interaction behavior [80]. Nevertheless, it is consistent with histological studies showing that peripheral administered fluorescent ghrelin does not reach the VTA [81]. One potential limitation is our use of photometry, as it measures population activity and cannot address differences in VTA DA subpopulations or individual VTA DA neurons. Nevertheless, the potentiated VTA dopamine activity to PB or chow presentation after ghrelin injection suggests an important role of indirect ghrelin sensitive pathway. While the nature of this input is unknown, AgRP^GHSR^ neurons are a strong possibility given the ability of AgRP neurons to influence VTA dopamine activity [76] and the attenuated dopamine release to ghrelin in AgRP GHSR KO mice in the current study. Other possibilities include an interaction with orexin neurons in the lateral hypothalamus since the effects of ghrelin on food reward are absent in orexin KO mice [32].

In summary, our results demonstrate that AgRP^GHSRs^ influence both AgRP neural activity and NAc dopamine release to the sensory detection of food and non-food objects. While ghrelin was always considered an important feedback signal to defend against weight loss and starvation [82], these results highlight the novel possibility that ghrelin signalling in AgRP neurons affects AgRP neuronal activity to the sensory detection of food and food cues. Thus, AgRP^GHSRs^ integrate an interoceptive energy state with current external sensory information to produce an optimal response in AgRP neural activity. In this manner, ghrelin signalling in AgRP neurons controls energy balance through a process of energy allostasis [1], in which the integration of energy need with the current sensory information of caloric information is likely to facilitate optimal behaviour when exposed to similar sensory cues in the future. This may be a novel principle in which neural hunger-sensing accelerates learning.

## Figures and Tables

**Figure:**
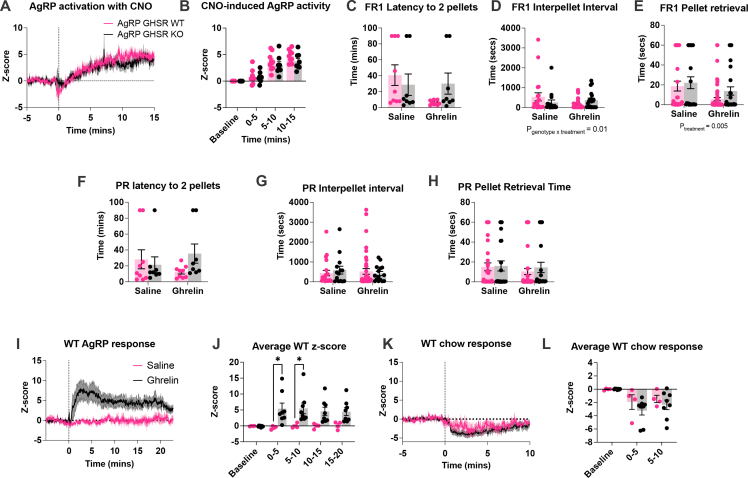
Supplementary figure 1: Deletion of GHSR from AgRP neurons does not affect DREADD stimulation of AgRP neurons. AgRP cre mice were co-injected with AAV9-hSyn-FLEX-GCaMP6s and AAV5-hSyn-DIO-hM3D(Gq)-mCherry to transduce AgRP neurons with both GCamP6s and hM3D(Gq). CNO increased average Z-score over time (A) and averaged time binned Z-scores (B) in both AgRP GHSR WT (n = 9) and KO mice (n = 8). Analysis of feeding behaviour in FR1 FED3 experiments, including latency to collect 2 pellets (C), FR1 interpellet interval (D) and pellet retrieval time (E). Analysis of feeding behaviour during PR FED3 experiments, including latency to collect 2 pellets (F), FR1 interpellet interval (G) and pellet retrieval time (H). Comparison of IP saline and ghrelin on AgRP activity in WT mice (I) with averaged time-binned Z-scores (J) (WT n = 4; KO n = 8). The AgRP response to chow introduction in WT mice after IP saline or ghrelin (K) with averaged time-binned Z-scores (L). Data +/− SEM. Dotted lines represents; A) time at injection of CNO; I) time of saline or ghrelin injection; K) time of chow introduction. Two-way ANOVA main effect of time (B,L) and main effect of genotype (L), main effect of treatment (E) and significant interaction between genotype and treatment (D). Post hoc Sidak's multiple comparisons (D); ∗p < 0.05. For a detailed description of statistics see statistical table 1.

**Figure:**
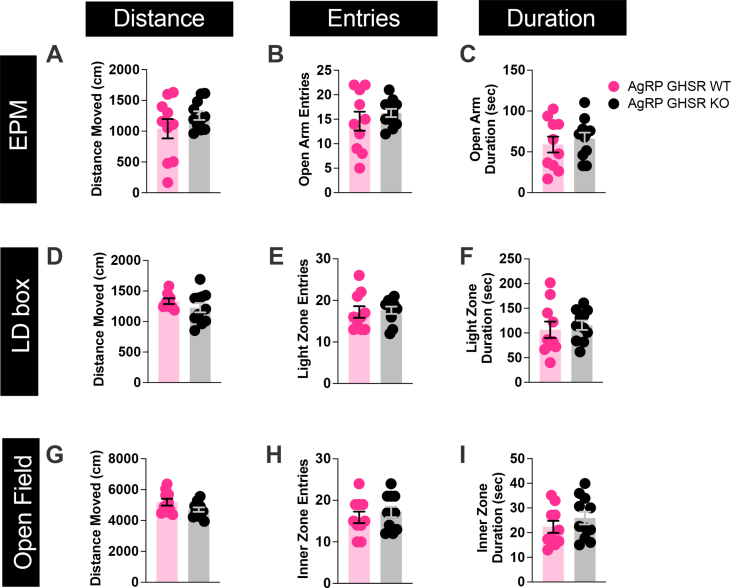
Supplement Figure 2: **Deletion of GHSRs in AgRP neurons does not affect anxiety-like behaviour in ad libitum fed mice.** Anxiety-like behaviour in ad libitum fed AgRP GHSR WT or AgRP GHSR KO mice was assessed using Elevated Plus Maze (EPM), Light Dark Box (LD Box) and Open Field test. In the EPM, no differences in distance moved (A), number of open arm entries (B) or time in the open arm (C) were observed. There were no differences in distance moved (D), number of light zone entries (E) or time in the light zone (F) in the LD Box test. Similar for the Open Field test, there were no differences in distance moved (G), number of inner zone entries (H) or time in the inner zone (I). Data +/− SEM; AgRP GHSR WT n = 10, AgRP GHSR KO n = 11; student's t-test. For a detailed description of statistics see statistical table 1.

**Figure:**
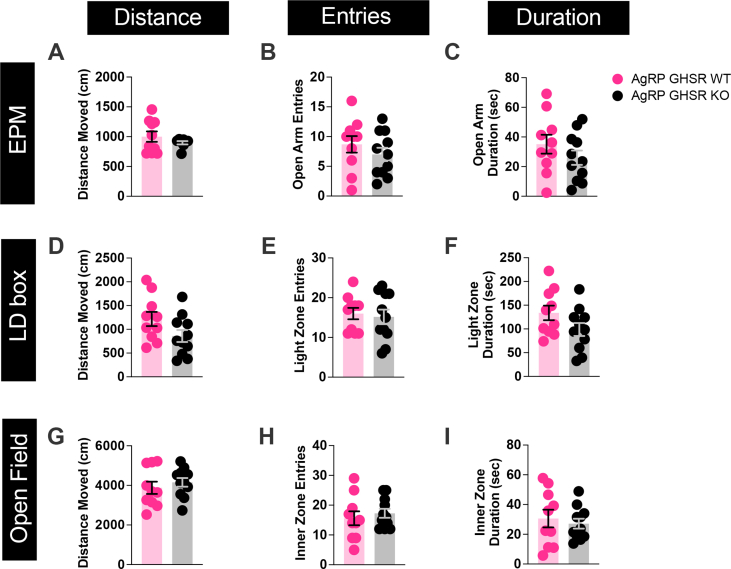
Supplement Figure 3: **Deletion of GHSRs in AgRP neurons does not affect anxiety-like behaviour in overnight fasted mice.** The Elevated Plus Maze (EPM), Light Dark Box (LD Box) and Open Field test were used to examine anxiety-like behaviour in overnight fasted (14–16 h) AgRP GHSR WT or AgRP GHSR KO mice. There were no differences in distance moved (A), number of open arm entries (B) or time in the open arm (C) of the EPM. In the LD Box, no differences in distance moved (D), number of light zone entries (E) or time in the light zone (F) were observed. Similar for the Open Field test, there were no differences in distance moved (G), number of inner zone entries (H) or time in the inner zone (I). Data +/− SEM; AgRP GHSR WT n = 10, AgRP GHSR KO n = 11; student's t-test. For a detailed description of statistics see statistical table 1.

**Figure:**
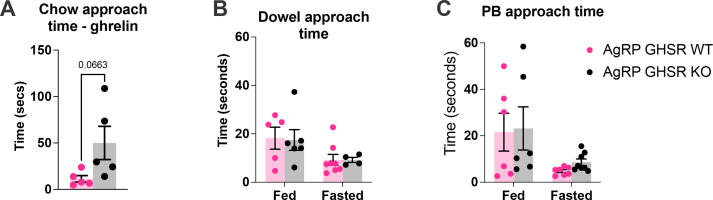
Supplementary Figure 4: Approach time during AgRP photometry sessions. (A) The time taken for mice to approach and contact chow food after ghrelin injection in WT and KO mice (WT n = 5, KO n = 5). Fasting significantly reduced the time mice required to approach and contact dowel (B) and PB (C; main effect of metabolic state), however no significant effects of genotype were observed (WT&KO n = 4–8). Students t-test (A) and two-way ANOVA. For a detailed description of statistics see statistical table 1.

**Figure:**
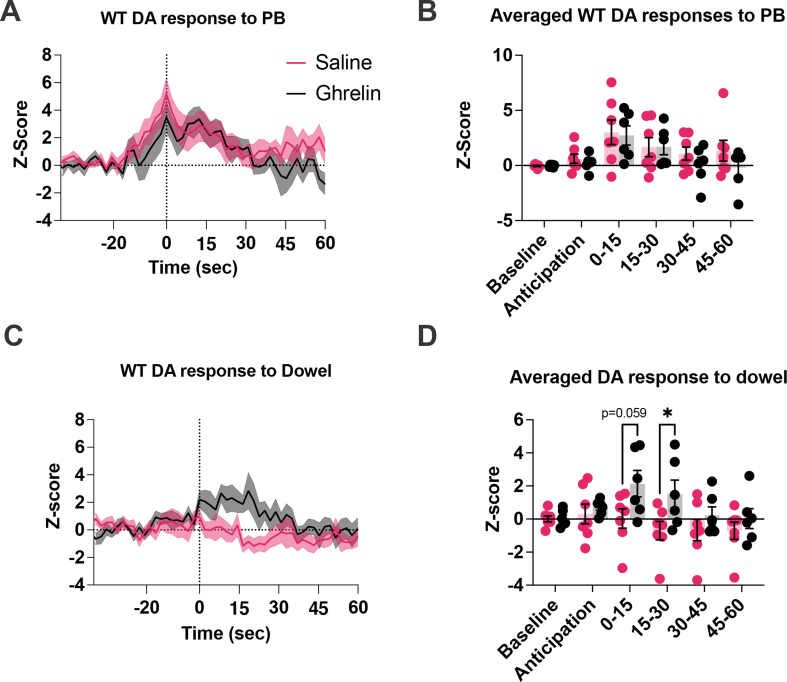
Supplementary Figure 5: GHSR-mediated NAc dopamine release in WT mice after IP saline of ghrelin. (A) NAc dopamine release at contact with PB in WT mice after IP saline and ghrelin. (B) Average time binned Z-score responses to PB contact in WT mice treated with IP saline or ghrelin (saline n = 7, ghrelin n = 6). (C) NAc dopamine release at contact with a wooden dowel in WT mice after IP saline and ghrelin. (D) Average time binned Z-score responses to PB contact in WT mice treated with IP saline or ghrelin (saline n = 7, ghrelin n = 6). Data +/− SEM. Dotted lines in A and C represent first contact to PB or wooden dowel. For a detailed description of statistics see statistical table 1. Two-way ANOVA with post hoc Sidak's multiple comparisons (D); ∗p < 0.05.

**Figure:**
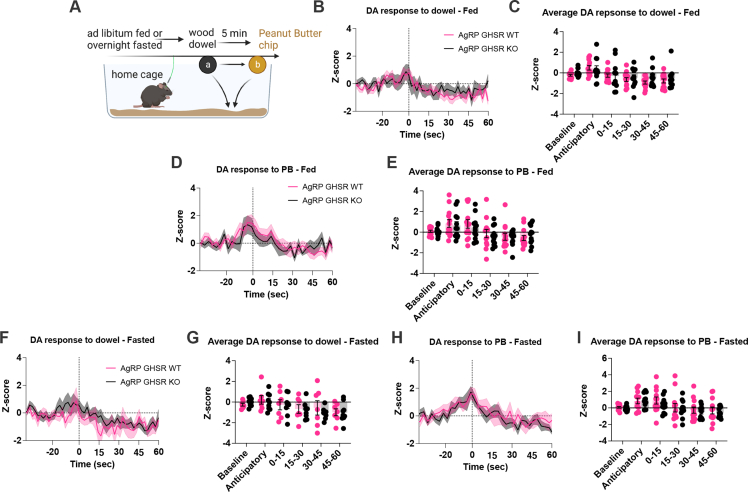
Supplementary Figure 6: Fasting did not reveal GHSR-mediated NAc dopamine release. (A) Schematic of experimental timeline created with BioRender.com where mice expressing GRAB-DA had ad-libitum access to chow or were fasted overnight before being introduced to a wooden dowel and peanut butter. Averaged Z-score traces in ad libitum fed mice aligned with point of first contact with a wooden dowel (B; WT n = 12, KO n = 13) and respective time averaged bins (C; WT n = 12, KO n = 13). Averaged Z-score traces aligned with point of first contact with peanut butter (D; WT n = 12, KO n = 13) and respective time averaged bins (E; WT n = 12, KO n = 13). Averaged Z-score traces in fasted mice aligned with point of first contact with a wooden dowel (F) and respective time averaged bins (G). Averaged Z-score traces with peanut butter (H) and respective time averaged bins (I). Data +/− SEM. Dotted lines in B, D, F, H represent first contact to wood dowel or PB. For a detailed description of statistics see statistical table 1.

## Data Availability

Data will be made available on request.
