# Supplementary material for: Ghrelin signalling in AgRP neurons links metabolic state to the sensory regulation of AgRP neural activity
Source: Mol Metab. 2023 Oct 26;78:101826. doi: 10.1016/j.molmet.2023.101826 (PMC10643323; doi:10.1016/j.molmet.2023.101826)
Supplement: Multimedia component 1 [file mmc1.pdf]

Table S1. Summary of statistical tests; animal studies

Figure	Statisitical test	n	p-value	Post test	p-value
**1B FR1**	**Two-way ANOVA**	WT=9, AgRP-GHSR-/-=8		Šidák multiple comparisons test	
	Interaction (F (1, 30) = 6.623)		**0.0153**	Saline: WT vs. Saline: KO	0.9999
	Treatment (F (1, 30) = 9.475)		**0.0044**	**Saline:WT vs. Ghrelin: WT**	**0.0016**
	Genotype (F (1, 30) = 6.338)		**0.0174**	Saline:KO vs. Ghrelin:KO	0.9996
				**Ghrelin:WT vs. Ghrelin:KO**	**0.0026**
**1C Progressive ratio**	**Two-way ANOVA**	WT=8-9, AgRP-GHSR-/-=9		Šidák multiple comparisons test	
	Interaction (F (1, 29) = 5.925)		0.0213	Saline: WT vs. Saline: KO	0.99
	Treatment (F (1, 29) = 2.944)		0.0969	**Saline:WT vs. Ghrelin: WT**	**0.0344**
	Genotype (F (1, 29) = 11.10)		**0.0024**	Saline:KO vs. Ghrelin:KO	0.997
				**Ghrelin:WT vs. Ghrelin:KO**	**0.0017**
**1J IP ghrelin averages**	**Two-way ANOVA**	WT=8, AgRP-GHSR-/-=8		Sidak's multiple comparison test	
	Interaction (F (4, 70) = 2.471)		0.0524	Baseline	>0.9999
	Treatment (F (4, 70) = 3.342)		**0.0146**	0-5	**0.0004**
	Genotype (F (1, 70) = 39.56)		**<0.0001**	5-10	**0.0021**
				10-15	**0.0147**
				15-20	**0.0238**
**1L IP ghrelin chow averages**	**Two-way ANOVA**	WT=8, AgRP-GHSR-/-=8		Sidak's multiple comparison test	
	Interaction (F (2, 42) = 2.913)		0.0654	Baseline	>0.9999
	Treatment (F (2, 42) = 13.69)		**<0.0001**	0-5	**0.0045**
	Genotype (F (1, 42) = 7.969)		**0.0072**	5-10	0.3611
**1M Weight consumed IP ghrelin**	**Unpaired two-tailed t test (t=2.373, (**	WT=8, AgRP-GHSR-/-=6	**0.0009**		
**1O IP saline averages**	**Two-way ANOVA**	WT=4, AgRP-GHSR-/-=4		Sidak's multiple comparison test	
	Interaction (F (4, 30) = 0.9261)		0.4619	Baseline	0.9999
	Treatment (F (4, 30) = 2.765)		**0.0455**	0-5	0.9986
	Genotype (F (1, 30) = 5.880)		**0.0216**	5-10	0.9791
				10-15	0.2108
				15-20	0.1761
**1Q IP saline chow averages**	**Two-way ANOVA**	WT=4, AgRP-GHSR-/-=4		Sidak's multiple comparison test	
	Interaction (F (2, 18) = 0.1961)		0.8236	Baseline	0.9986
	Treatment (F (2, 18) = 8.64)		**0.0023**	0-5	0.9773
	Genotype (F (1, 18) = 0.3081)		0.5857	5-10	0.8514
**2C Wooden dowel fed averages**	**Two-way ANOVA**	WT=8, AgRP-GHSR-/-=4		Sidak's multiple comparison test	
	Interaction (F (3, 40) = 0.5346)		0.6612	Baseline	0.9989
	Time (F (3, 40) = 0.5776)		0.6331	Anticipatory	0.803
	Genotype (F (1, 40) = 5.573)		**0.0232**	0-60 secs	0.3278
				0-120 secs	0.2827
**2E Peanut butter fed averages**	**Two-way ANOVA**	WT=7, AgRP-GHSR-/-=8		Sidak's multiple comparison test	
	Interaction (F (3, 52) = 1.028)		0.3877	Baseline	0.9994
	Time (F (3, 52) = 24.29)		**<0.0001**	Anticipatory	0.9857
	Genotype (F (1, 52) = 6.701)		**0.0229**	0-60 secs	0.09
				0-120 secs	0.1252
**2H Wooden dowel fasted averages**	**Two-way ANOVA**	WT=8, AgRP-GHSR-/-=4		Sidak's multiple comparison test	
	Interaction (F (3, 40) = 2.505)		0.0857	Baseline	0.9992
	Time (F (3, 40) = 6.498)		**0.0038**	Anticipatory	0.8437
	Genotype (F (1, 40) = 14.57)		**0.0007**	0-60 secs	**0.01**
				0-120 secs	**0.0084**
**2J Peanut butter fasted averages**	**Two-way ANOVA**	WT=8, AgRP-GHSR-/-=8		Sidak's multiple comparison test	
	Interaction (F (3, 56) = 4.882)		**0.0044**	Baseline	0.9997
	Time (F (3, 56) = 24.66)		**<0.0001**	Anticipatory	0.9997
	Genotype (F (1, 56) = 17.29)		**0.0001**	0-60 secs	**0.0007**
				0-120 secs	**0.0008**
				1-2	0.001
**3C DA response - IP saline dowel av**	**Two-way ANOVA**	WT=7, AgRP-GHSR-/-=6		Sidak's multiple comparison test	
	Interaction (F (5, 66) = 0.2260)		0.95	Baseline	>0.9999
	Time (F (5, 66) = 0.9563)		0.451	Anticipation	0.9878
	Genotype (F (1, 66) = 2.169)		0.1455	0-15	>0.9999
				15-30	0.7716
				30-45	0.9531
				45-60	0.9767
**3E DA response - IP ghrelin dowel a**	**Two-way ANOVA**	WT=6, AgRP-GHSR-/-=6		Sidak's multiple comparison test	
	Interaction (F (5, 60) = 1.406)		0.235	Baseline	>0.9999
	Time (F (5, 60) = 2.437)		**0.0447**	Anticipation	>0.9999
	Genotype (F (1, 60) = 6.445)		**0.0137**	0-15	0.2375
				15-30	**0.0464**
				30-45	0.7796
				45-60	>0.9999
**3G DA response - IP saline PB avera**	**Two-way ANOVA**	WT=7, AgRP-GHSR-/-=6		Sidak's multiple comparison test	
	Interaction (F (5, 66) = 0.9728)		0.441	Baseline	>0.9999
	Time (F (5, 66) = 2.566)		**0.035**	Anticipation	>0.9999
	Genotype (F (1, 66) = 8.075)		**0.006**	0-15	0.2248
				15-30	0.8733
				30-45	0.4628
				45-60	0.1889
**3I DA response - IP ghrelin PB avera**	**Two-way ANOVA**	WT=6, AgRP-GHSR-/-=6		Sidak's multiple comparison test	
	Interaction (F (5, 60) = 1.566)		0.1834	Baseline	>0.9999
	Time (F (5, 60) = 4.527)		**0.0014**	Anticipation	>0.9999

	Genotype (F (1, 60) = 6.174)		**0.0158**	0-15	**0.0292**
				15-30	0.169
				30-45	0.9998
				45-60	0.9845
**4C VTA DA response to ip ghrelin**	**Two-way ANOVA**	WT=6, AgRP-GHSR-/-=6		Sidak's multiple comparison test	
	Interaction (F (3, 24) = 1.566)		0.5918	Baseline	0.888
	Time (F (3, 24) = 4.527)		0.3136	0-15	0.9968
	Genotype (F (1, 24) = 6.174)		0.4996	5-10	0.9932
				10-15	0.6495
**4E VTA DA PB max Z-score**	**Unpaired t test** (t=7.475, df=12)	WT=8, AgRP-GHSR-/-=6	**<0.0001**		
**4G VTA DA chow averages**	**Two-way ANOVA**	WT=4, AgRP-GHSR-/-=4		Šídák's multiple comparisons test	
	Interaction (F (1, 12) = 6.755)		**0.0233**	baseline	0.9933
	Time (F (1, 12) = 1.266)		0.2824	chow	**0.0052**
	Genotype (F (1, 12) = 7.548)		**0.0177**		
**S1B AgRP activation with CNO avera**	**Two-way ANOVA**	WT=9, AgRP-GHSR-/-=8		Šídák's multiple comparisons test	
	Interaction F (3, 60) = 0.6348;		0.5955	Baseline	>0.9999
	Time F (3, 60) = 44.17;		**<0.0001**	0-5	>0.9999
	Genotype F (1, 60) = 1.934		0.1695	5-10	0.7692
				10-15	0.3478
**S1C FR1 latency to 2 pellets**	**Two-way ANOVA**	WT=9, AgRP-GHSR-/-=8		Šídák's multiple comparisons test	
	Interaction F (1, 30) = 2.572		0.1193	WT-KO Saline	0.703
	Treatment F (1, 30) = 0.1489		0.1489	WT-KO Ghrelin	0.2625
	Genotype F (1, 30) = 0.2844		0.5977		
**S1D FR1 Interpellet Interval**	**Two-way ANOVA**	WT saline n=20, WT ghrelin n=56		Šídák's multiple comparisons test	
	Interaction F (1, 106) = 6.788	KO saline n=16, KO ghrelin n=20	**0.0106**	WT-KO Saline	0.1619
	Treatment F (1, 30) = 2.017	from WT=9, AgRP-GHSR-/-=8 mice	0.1584	WT-KO Ghrelin	0.0944
	Genotype F (1, 30) = 0.2077		0.8857		
**S1E FR1 Pellet Retrieval Time**	**Two-way ANOVA**	WT saline n=27, WT ghrelin n=62		Šídák's multiple comparisons test	
	Interaction F (1, 135) = 0.4155	KO saline n=22, KO ghrelin n=28	0.5203	WT-KO Saline	0.8172
	Treatment F (1, 135) = 8.110	from WT=9, AgRP-GHSR-/-=8 mice	**0.0051**	WT-KO Ghrelin	0.1575
	Genotype F (1, 135) = 2.345		0.128		
**S1F PR latency to 2 pellets**	**Two-way ANOVA**	WT=9, AgRP-GHSR-/-=8		Šídák's multiple comparisons test	
	Interaction F (1, 30) = 2.33		0.1371	WT-KO Saline	0.8594
	Treatment F (1, 30) = 0.0093;		0.9237	WT-KO Ghrelin	0.2009
	Genotype F (1, 30) = 1.934		0.6872		
**S1G PR Interpellet Interval**	**Two-way ANOVA**	WT saline n=21, WT ghrelin n=40		Šídák's multiple comparisons test	
	Interaction F (1, 86) = 0.5477	KO saline n=14, KO ghrelin n=15	0.4613	WT-KO Saline	0.812
	Treatment F (1, 86) = 0.0646	from WT=9, AgRP-GHSR-/-=8 mice	0.7999	WT-KO Ghrelin	0.8721
	Genotype F (1, 86) = 0.0156		0.9006		
**S1H PR Pellet Retrieval Time**	**Two-way ANOVA**	WT saline n=30, WT ghrelin n=49		Šídák's multiple comparisons test	
	Interaction F (1, 118) = 0.1377	KO saline n=22, KO ghrelin n=21	0.7112	WT-KO Saline	0.9865
	Treatment F (1, 118) = 0.5038	from WT=9, AgRP-GHSR-/-=8 mice	0.4792	WT-KO Ghrelin	0.7332
	Genotype F (1, 118) = 0.3433		0.5591		
**S1J WT AgRP activation IP saline vs**	Interaction F (4, 50) = 1.511	Saline n=4, Ghrelin n=8	0.2131	Šídák's multiple comparisons test	
	Time F (4,50) = 1.38		0.2515	Baseline	>0.9999
	Genotype F (1, 50) = 21.50		**<0.0001**	0-5	**0.0184**
				5-10	**0.0343**
				10-15	0.113
				15-20	0.1478
**S1L WT AgRP responses to chow**	Interaction F (2, 30) = 0.568	Saline n=4, Ghrelin n=8	0.6075	Šídák's multiple comparisons test	
	Time F (2, 30) = 7.931		**<0.0017**	Baseline	>0.9999
	Genotype F (1, 30) = 1.693		0.2031	0-5	0.4602
				5-10	0.7354
**S2A EPM FED Distance**	t=1.270, df=19	WT=10, AgRP-GHSR-/-=11	0.2193		
**S2B EPM FED Open Arm Entries**	t=0.8215, df=19	WT=10, AgRP-GHSR-/-=11	0.4215		
**S2C EPM FED Open Arm Duration**	t=0.6202, df=19	WT=10, AgRP-GHSR-/-=11	0.5425		
**S2D LD Box FED Distance**	t=1.078, df=19	WT=10, AgRP-GHSR-/-=11	0.2963		
**S2E LD Box FED Light Zone Entries**	t=0.2699, df=19	WT=10, AgRP-GHSR-/-=11	0.7901		
**S2F LD Box FED Light Zone Duratic**	t=0.4886, df=19	WT=10, AgRP-GHSR-/-=11	0.6307		
**S2G OF FED Distance**	t=1.880, df=19	WT=10, AgRP-GHSR-/-=11	0.0756		
**S2H OF FED Inner Zone**	t=0.6889, df=19	WT=10, AgRP-GHSR-/-=11	0.4992		
**S2I OF FED Inner Zone Duration**	t=0.9975, df=19	WT=10, AgRP-GHSR-/-=11	0.3311		
**S3A EPM FASTED Distance**	t=1.025, df=16	WT=10, AgRP-GHSR-/-=8	0.3205		
**S3B EPM FASTED Open Arm Entries**	t=0.9631, df=19	WT=10, AgRP-GHSR-/-=11	0.3476		
**S3C EPM FASTED Open Arm Duratic**	t=1.150, df=19	WT=10, AgRP-GHSR-/-=11	0.2643		
**S3D LD Box FASTED Distance**	t=1.840, df=19	WT=10, AgRP-GHSR-/-=11	0.0814		
**S3E LD Box FASTED Light Zone En**	t=0.3475, df=19	WT=10, AgRP-GHSR-/-=11	0.732		
**S3F LD Box FASTED Light Zone Du**	t=1.712, df=19	WT=10, AgRP-GHSR-/-=11	0.1031		
**S3G OF FASTED Distance**	t=0.7326, df=19	WT=10, AgRP-GHSR-/-=11	0.4727		
**S3H OF FASTED Inner Zone**	t=0.6149, df=19	WT=10, AgRP-GHSR-/-=11	0.5459		
**S3I OF FASTED Inner Zone Duratio**	t=0.5169, df=19	WT=10, AgRP-GHSR-/-=11	0.6112		
**S4A Chow approach time**	t=2.125, df=8	WT=5, AgRP-GHSR-/-=5	0.0663		
**S4B Dowel Approach Time**	**Two-way ANOVA**	WT n = 5-8 (fed/fasted)		Šídák's multiple comparisons test	
	Interaction F (1, 19) = 0.0096	KO n = 4-6 (fed/fasted)	0.9228	WT-KO Fed	0.9856
	Treatment F (1, 19) = 6.015		0.024	WT-KO Fasted	0.9999

	Genotype F (1, 19) = 0.0136		0.9082		
**S4PB PB Approach Time**	**Two-way ANOVA**	WT n = 5-8 (fed/fasted)		Šídák's multiple comparisons test	
	Interaction F (1, 23) = 0.0405	KO n = 4-6 (fed/fasted)	0.8423	WT-KO Fed	0.9768
	Treatment F (1, 23) = 7.953		**0.0097**	WT-KO Fasted	0.8472
	Genotype F (1, 23) = 0.2409		0.6282		
**S5B DA response - PB fed averages**	**Two-way ANOVA**	Saline n=7, Ghrelin n=6		Šídák's multiple comparisons test	
	Interaction (F (5,66) = 0.3796)		0.861	Baseline	>0.9999
	Time (F (5,66) = 4.525)		**0.0013**	Anticipatory	0.9993
	Genotype (F (1, 66) = 1.70)		0.1968	0-15	0.9999
				15-30	>0.9999
				30-45	0.8883
				45-60	0.5766
**S5D DA response - dowel fed averag**	**Two-way ANOVA**	Saline n=7, Ghrelin n=6		Šídák's multiple comparisons test	
	Interaction (F (5,66) = 1.285)		0.2812	Baseline	>0.9999
	Time (F (5,66) = 1.797)		0.1255	Anticipatory	0.9964
	Genotype (F (1, 66) = 10.76)		**0.0017**	0-15	0.0593
				15-30	**0.0404**
				30-45	0.8458
				45-60	0.9286
**S6C DA response - dowel fed averag**	**Two-way ANOVA**	WT=12, AgRP-GHSR-/-=11		Šídák's multiple comparisons test	
	Interaction (F (5,130) = 0.4097)		0.8414	Baseline	0.9556
	Time (F (5,130) = 6.657)		**<0.0001**	Anticipatory	0.9986
	Genotype (F (1, 130) = 1.413)		0.2367	0-15	>0.9999
				15-30	0.9957
				30-45	0.7434
				45-60	0.9603
**S6E DA response - PB fed averages**	**Two-way ANOVA**	WT=12, AgRP-GHSR-/-=13		Šídák's multiple comparisons test	
	Interaction (F (5,138) = 0.3760)		0.8645	Baseline	>0.9999
	Time (F (5,138) = 5.953)		**<0.0001**	Anticipatory	0.9985
	Genotype (F (1, 138) = 0.3380)		0.562	0-15	0.9265
				15-30	0.9896
				30-45	>0.9999
				45-60	0.9529
**S6G DA response - dowel fasted ave**	**Two-way ANOVA**	WT=7, AgRP-GHSR-/-=9		Šídák's multiple comparisons test	
	Interaction (F (5,84) = 0.2557)		0.9358	Baseline	0.9966
	Time (F (5,84) = 3.295)		**0.0091**	Anticipatory	0.9996
	Genotype (F (1, 84) = 0.2746)		0.6016	0-15	>0.9999
				15-30	>0.9999
				30-45	0.8801
				45-60	>0.9999
**S6I DA response - PB fasted average:**	**Two-way ANOVA**	WT=12, AgRP-GHSR-/-=12		Šídák's multiple comparisons test	
	Interaction (F (5,132) = 0.2346)		0.9467	Baseline	>0.9999
	Time (F (5,132) = 5.916)		**<0.0001**	Anticipatory	>0.9999
	Genotype (F (1, 132) = 1.224)		0.2706	0-15	0.783
				15-30	0.9998
				30-45	0.9937
				45-60	0.9881
